# Making the Invisible Visible: Verbal but Not Visual Cues Enhance Visual Detection

**DOI:** 10.1371/journal.pone.0011452

**Published:** 2010-07-07

**Authors:** Gary Lupyan, Michael J. Spivey

**Affiliations:** 1 University of Pennsylvania, Philadelphia, Pennsylvania, United States of America; 2 University of California Merced, Merced, California, United States of America; Kyushu University, Japan

## Abstract

**Background:**

Can hearing a word change what one sees? Although visual sensitivity is known to be enhanced by attending to the location of the target, perceptual enhancements of following cues to the identity of an object have been difficult to find. Here, we show that perceptual sensitivity is enhanced by verbal, but not visual cues.

**Methodology/Principal Findings:**

Participants completed an object detection task in which they made an object-presence or -absence decision to briefly-presented letters. Hearing the letter name prior to the detection task increased perceptual sensitivity (*d*′). A visual cue in the form of a preview of the to-be-detected letter did not. Follow-up experiments found that the auditory cuing effect was specific to validly cued stimuli. The magnitude of the cuing effect positively correlated with an individual measure of vividness of mental imagery; introducing uncertainty into the position of the stimulus did not reduce the magnitude of the cuing effect, but eliminated the correlation with mental imagery.

**Conclusions/Significance:**

Hearing a word made otherwise invisible objects visible. Interestingly, seeing a preview of the target stimulus did not similarly enhance detection of the target. These results are compatible with an account in which auditory verbal labels modulate lower-level visual processing. The findings show that a verbal cue in the form of hearing a word can influence even the most elementary visual processing and inform our understanding of how language affects perception.

## Introduction

To what extent can high-level cognitive expectation influence low-level sensory processing? Allocating visual attention to a location improves reaction times (RTs) to probes appearing in that location [1]. The spread of attention is also affected by specific objects: cuing an object speeds responses to a probe within the cued object's boundaries, e.g., [2], [3].

There is now accumulating evidence that higher level semantic information can influence visual perception in some surprising ways. For instance, auditory processing of verbs associated with particular directions of motion (e.g., fly, bomb) interferes with visual discrimination tasks along the vertical axis [4] and increases sensitivity to the congruent motion direction in random-dot kinematograms [5]. Moreover, linguistic input can guide visual search in an incremental and automatic fashion [6], [7]. Ascribing meaning to unfamiliar shapes using verbal labels improves the efficiency of visual search for these shapes [8]. In fact, simply hearing a word that labels the target improves the speed and efficiency of search (compared to not hearing the label, but still knowing the target's identity). For instance, when searching for the number 2 among 5's, participants are faster to find the target when they actually hear “find the two” immediately prior to the search trial [9] – even when they know that the 2 is the target because is has been so for the entire block of trials. Such facilitation of visual processing by verbal labels is disrupted by manipulations that preserve the low-level visual features of a stimulus but alter its association with the named category (e.g., through a mirror reversal) [10].

Although it is now well-established that spatial cues can modulate perceptual sensitivity (independent of decision bias) in visual detection tasks [11]–[13], the efficacy of cues to non-spatial attributes such as shape and color on perceptual sensitivity remains controversial, e.g., [14]. The efficacy of information from outside vision (e.g., verbal cues) to alter visual sensitivity is even less explored. In the present work, we test whether hearing object names improves participants' sensitivity (*d′*) in detecting the presence of a single briefly presented visual object—a task that does not require a search process, nor explicit identification or categorization of the stimulus.

Perception researchers have long exploited signal detection measures as a way to distinguish between two presumed stages involved in responding to perceptual stimuli: 1) a sensory detection stage, where the physical similarity between a “noise trial” and a “signal+noise trial” can be determined as a measure of sensitivity, or *d′*, and 2) a decision stage, where higher-level interpretation and cognitive processes invariably include a response bias that can be determined as a measure of *ß* or *c* [15]–[17]. In using *d′* as our dependent measure, we are able to dissociate changes in sensitivity from changes in response/decision bias. See [12] for a demonstration of why a change in *d′* cannot be produced by a simple change in the decision bias.

Many prior demonstrations of attention on visual processing have relied on mean RTs as the dependent measure making it difficult or impossible to tease apart early-stage effects (e.g., object detection) from late-stage effects (e.g., object recognition). This is not to say that it is impossible to use RT measures to discriminate between perceptual and decisional. For example, Sigman and Dehaene [18] use distributional analyses of RTs in cognitive tasks to separately analyze perceptual, decision, and response stages of processing [see also 19]. For example, although it is well established that RTs to identify objects can be improved through previous exposure to the objects [20], [21], such mean RT measures do not distinguish whether the improvement results from a decision-level process (traditional priming accounts), or through genuine facilitation of perceptual processes cf. [22], [23]. Thus although there is accumulating evidence of linguistic effects on performance in perceptual tasks, there is at present insufficient evidence to conclude that hearing verbal labels alters early visual processing.

The hypothesis guiding the present work is that a linguistic facilitation of visual processing arises due to an interaction between different sources of sensory evidence taking place when two different sensory modalities provide non-overlapping forms of support for the same perceptual category [24], [25]. In terms of a normative treatment of statistical evidence, the mutual interaction between two sensory inputs (e.g., auditory cues for a visual task) should actually be more effective than when the same sensory modality provides two non-independent sources of sensory evidence (e.g., visual cues for a visual task). The present study uses signal detection theory to test specifically for an enhancement of visual perceptual sensitivity conferred by auditory linguistic cues, as compared to that conferred by visual cues. We find that congruent auditory linguistic cues, but not visual cues, significantly improve perceptual sensitivity (as separate from decision bias) for detecting the presence of a visual stimulus. We then investigate the extent of these effects through follow-up studies.

## Results and Discussion

### Experiment 1

In the first experiment we test our central prediction that a cue, particularly a linguistic cue, presented prior to a simple detection task will improve the detection sensitivity of the labeled stimuli. The decision in the present experiments is simply “present” vs. “absent.” The identity of the to-be-detected (target) stimulus, although occasionally consciously perceived, is irrelevant to the task. A finding of greater *d′* on cued trials would constitute evidence of verbal cues improving basic visual processing.

**Figure 1:**
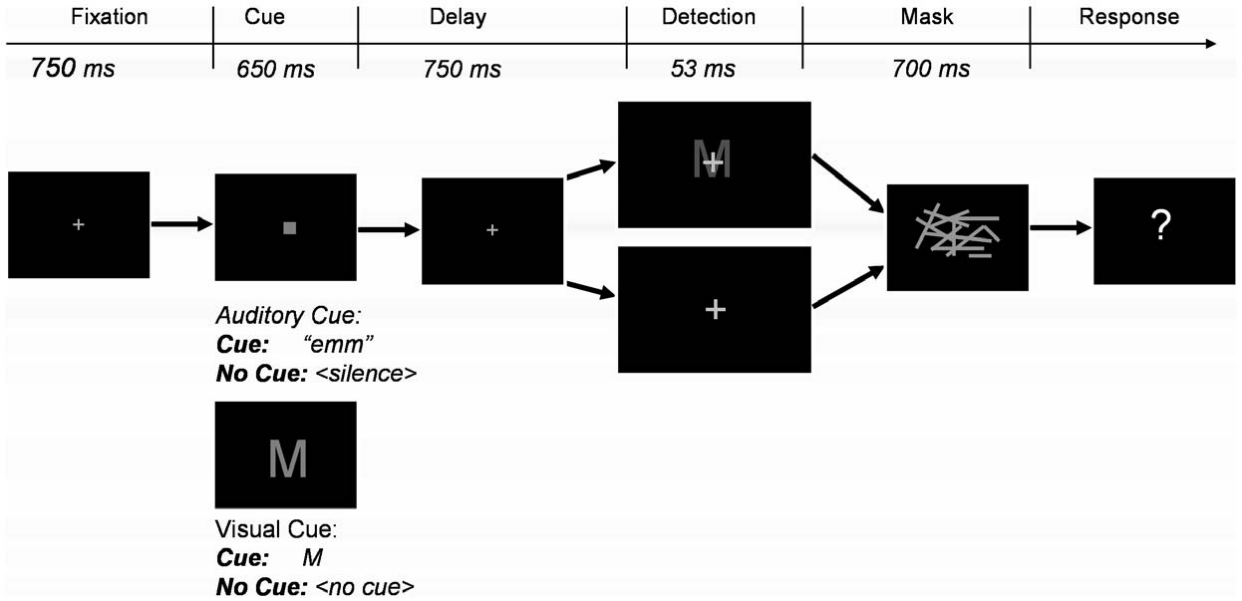
Trial structure of the basic cued object detection paradigm (Experiment 1). During the response part of the trial, participants respond *present* or *absent* depending on whether they detected a letter.

The participants' task was to detect the presence of briefly-flashed uppercase letters (Figure 1 outlines the basic design). Participants in the auditory-cue condition heard the name of the letter on 50% of the trials, informing them of the identity of the target letter. Participants in the visual-cue condition saw a visual preview of the target letter. In all cases, the cue did not predict target-presence.

**Figure 2:**
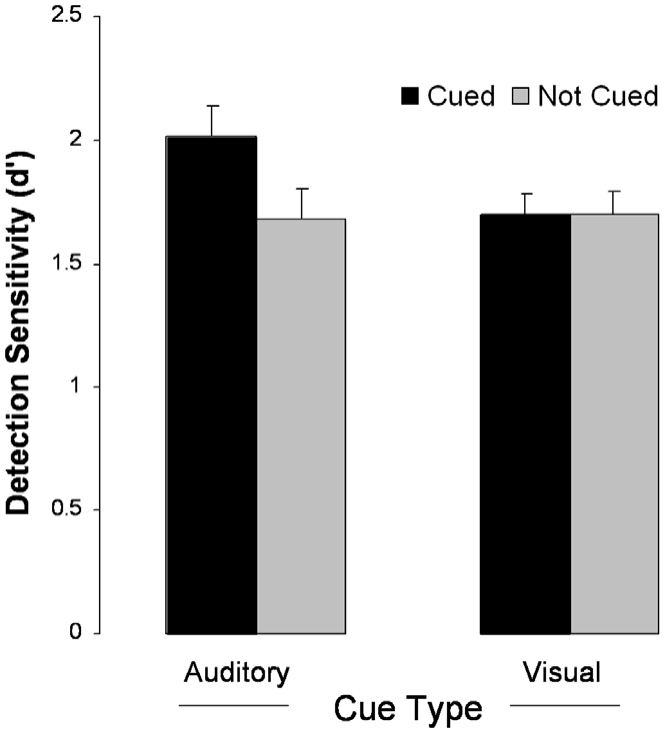
Results from Experiment 1 indicating effects of auditory and visual cues on the detection of cued visual objects. Bars indicate ±1 SE of the difference between the means.

Summary statistics for all experiments are presented in Tables 1–5. Hit rates on cued trials were significantly greater than hit rates on non-cued trials, *t*(19) = 3.68, *p* = .002 (Table 1). We computed *d′* in each condition in the standard way, by subtracting z-transformed false alarm rates from the z-transformed hit rates. For example, *d′* for the cued trials is given by *z*(Hits_cued_)–*z*(False-Alarms_cued_). Paired t-tests on the individual d′ values showed that sensitivity was significantly improved by auditory cues, *t*(19) = 2.37, *p* = .028 (Figure 2-left), but not by visual cues (Figure 2-right), *t*(20)<1. This difference in cuing efficacy was reflected in a significant cue-type×cue-presence interaction, *t*(40) = 2.22, *p* = .032.

**Table 1:** Summary statistics for Experiment 1.

Condition	Hits	FA	*d′* §	*natural log β*	*normalized criterion*
*Auditory Cues*					
Cued	.69 (.06)	.15 (.03)	2.1 (.36)	.58 (.36)	−.75 (.54)
No Cued	.59 (.06)	.11 (.02)	1.8 (.32)	.73 (.26)	.09 (.26)
Cohen's d*	d = .83	d = .47	d = .53		
Significance level of the difference	***p*** ** = .002**	*p* = .053	***p*** ** = .028**	*p* = .49	p = .15
*Visual Cues*					
Cued	.60 (.05)	.18 (.05)	1.76 (.34)	1.22 (.27)	.59 (.34)
No Cued	.57 (.05)	.16 (.05)	1.76 (.34)	1.35 (.29)	.53 (.68)
Significance level of the difference	*p* = .27	*p* = .25	*p* = .83	*p* = .47	*p* = .93

Condition Means (between-subject SEs).

*Effect sizes report Cohen's d (using the SD of the condition difference) and the *t*-value from a paired *t*-test between the values for cued and not-cued trial-types.

§ Separate *d′* values were computed for each subject. Following convention, false alarms of 0 and hits of 1 (both rare) were replaced by substituting 2n for n trials. Here, this translates to values of 1/200 and 199/200, respectively.

**Table 2 pone-0011452-t002:** Summary statistics for Experiment 2.

Condition	Hits	FA**	*d′* ^§^	*natural log β*	*normalized criterion*
Validly Cued	.73 (.05)	.25 (.06)	1.69 (.20)	.19 (55)	.07 (.24)
Invalidly Cued	.64 (.07)	**	1.43 (.28)	.26 (.53)	.20 (.24)
Not Cued	.52 (.06)	.20 (.06)	1.25 (.32)	.99 (.43)	.61 (.19)
valid vs. invalid	d = .75	¤	d = .76	¤	¤
	***p*** ** = .046**		***p*** ** = .039**		
valid vs. not cued	d = .89	**	d = .98		
	***p*** ** = .020**		***p*** ** = .013**	*p* = .25	*p* = .12
not cued vs. invalid	*p* = .16	*p* = .41	*p* = .13	*p* = .29	*p* = .25

Condition Means (between-subject SEs).

**Experiment 2 contained three trial types: validly cued, invalidly cued, and not cued. Separate false alarms cannot be computed for valid versus invalid trials, as the validity distinction collapses for target-absent trials. Hence, the reported p-value for False-Alarms corresponds to the cued versus non-cued trials.

¤ Because separate false alarms cannot be computed for valid versus invalid trials, any differences in the criterion between the validly and invalidly-cued trials would be artifactual.

**Table 3 pone-0011452-t003:** Summary statistics for Experiment 3.

Condition	Hits	FA	*d′* ^§^	*natural log β*	*normalized criterion*
Cued	.66 (.03)	.21 (.04)	1.67 (.30)	.92 (.24)	.17 (.07)
Not Cued	.56 (.04)	.19 (.04)	1.43 (.28)	1.11 (.27)	.37 (.23)
Cohen's d	d = .61		d = .50		
Cuing effect	**p = .013**	*p* = .39	***p*** ** = .036**	*p* = .11	p = .37

Condition Means (between-subject SEs).

**Table 4 pone-0011452-t004:** Summary statistics for Experiment 4.

Condition	Hits	FA	*d′* ^§^	*natural log β*	*normalized criterion*
Cued	.60 (.06)	.16 (.04)	1.68 (.26)	.98 (.25)	.71 (.42)
Not Cued	.47 (.06)	.13 (.03)	1.26 (.24)	.75 (.17)	1.89 (.65)
Cohen's d	d = .76		d = .67		
Cuing Effect	***p*** ** = .003**	*p* = .28	***p*** ** = .007**	*p* = .31	p = .15

Condition Means (between-subject SEs).

**Table 5:** Summary statistics for Experiment 5.

Condition	Hits	FA	*d′* ^§^	*natural log β*	*normalized criterion*
Cued	.57 (.06)	.32 (.07)	1.00 (.32)	.62 (.30)	.66 (.77)
Not Cued	.52 (.05)	.26 (.06)	1.01 (.30)	.88 (.31)	.02 (.97)
Cuing Effect	p = .24	p = .14	p = .63	p = .21	p = .49

Condition Means (between-subject SEs).

In addition to an auditory cuing effect on *d′*, we also observed a reduction in RTs from *M* = 476 ms to *M* = 434 ms, *t*(19) = 3.01, *p* = .007 (RTs included correct responses; trials with latencies above 2500 ms (3.3%) were excluded). There was no corresponding decrease in RTs in the visual condition, *F*<1. The effect of auditory cues on RTs is somewhat surprising considering that participants had 700 ms during the masking period in which to prepare their responses. The RT reduction likely reflects a blend of sensitivity and response bias (e.g., greater confidence in the response on cued trials). The *d′* difference demonstrates that hearing the name of the target letter significantly increased participants' sensitivity in detecting the anticipated letter. Individual RT differences were uncorrelated with individual magnitudes of the cuing effect, *r*<.1. In contrast to differences in *d′*, there were no observed differences in criterion as measured by natural-log ß and normalized *c* [17] for this or subsequent experiments (see Table 1).

This result is the first to demonstrate improvement in simple detection of a cued object. There is, of course, much evidence that cuing simple visual attributes such as color and direction of motion results in more efficient processing of the cued attributes [26]. The literature on cross-modal priming finds mixed evidence for facilitation of visual processing of objects following auditory cues. Existing evidence has focused on identification rather than detection tasks [27], [28]. However, there are reliable effects of spatial auditory cues on visual processing [29]. Störmer et al., [30] showed that modulation of visual cortex following laterally presented auditory cues occur within 100 ms of target onset. There is also evidence that informative cues can speed visual discrimination of cued and un-cued objects [31]. For example, Iordanescu et al. [32] showed that sounds characteristic of target objects such as the jingling of keys facilitates visual search for the associated objects in a visual search task. The task used in the present work contrasts with the relatively complex tasks used in the above studies. Our simple detection task required neither identification, selection, nor discrimination of target stimuli, though participants did need categorize each trial as an instance of “noise” (just the mask) or “signal+noise” (mask plus letter). Our measurement was perceptual sensitivity rather than reaction time (which may reflect contributions of decision bias). The present work is thus the first to show that auditory object labels can improve detection sensitivity of a basic visual process.

### Experiment 2

The finding of greater detection sensitivity on cued trials is subject to several confounds. First, it is possible that detection ability is improved simply by the attentional arousal induced by auditory stimulation. For example, it may be that hearing sounds produces a transient improvement in performance by increasing vigilance e.g., [33], although such effects generally require synchronous presentation [34]. An additional limitation of Experiment 1 is that the cues always validly predicted the target stimulus. Although the cues did not predict stimulus-presence, the cue and stimulus always matched on cued stimulus-present trials. It is thus not clear whether the cue needs to be valid to facilitate simple detection. The goal of Experiment 2 was to assess the specificity of the cuing effect by contrasting valid cues (those that matched the target stimulus) with invalid cues (those that did not match the target stimulus). As before, the cues did not predict stimulus-presence.

Experiment 2 was procedurally identical to the auditory condition Experiment 1 with the exception that the cued stimulus-present trials were evenly divided into cue-valid and cue-invalid trials. On invalid trials, the identity of the letter-cue did not match the target stimulus. Participants were told that “the cue would sometimes predict the identity of the target letter.”

**Figure 3:**
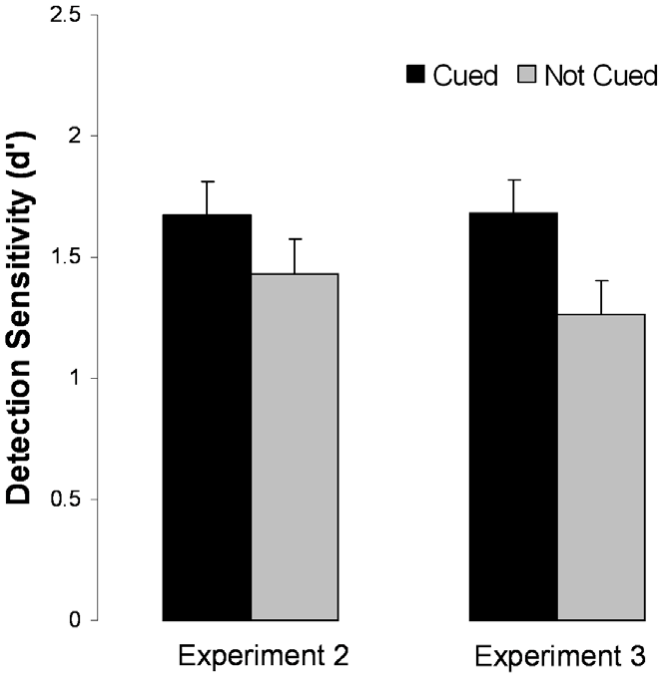
Results from Experiment 2. Bars indicate ±1 SE of the within-subject difference in the means. Asterisks indicate significant differences between condition means at p<.05.

Only valid cues improved detection sensitivity (Figure 3-right). Planned comparisons showed that sensitivity (*d′*) was significantly higher in valid trials than invalid trials, *t*(9) = 2.41, *p* = .039 (Table 2). A comparison of valid and no-cue trials once again revealed a significant advantage for the former, *t*(9) = 3.10, *p* = .013. There was no significant difference between invalid and no-cue trials, *t*(9) = 1.65, *p* = .13. As in Experiment 1, the difference in *d′* arose from differences in hit rates. Paired t-tests of hit-rates mirrored the *d′* analysis. There were no reliable RT effects.

Detection sensitivity was improved only when the auditory cues matched the to-be-detected (target) stimulus (validly-cued trials). This result further supports the hypothesis that auditory verbal labels have a facilitatory effect on the subsequent visual detection of single objects matching the verbal label.

### Experiments 3–4

One way in which auditory cues may have facilitated object detection is by encouraging participants to actively image the named letter. Such imagery strategies have been shown to improve detection performance to targets having contours that overlap with the imaged contours [35]. Detection enhancements due to mental imagery thus appear to be highly position dependent. If auditory cues facilitate object detection by encouraging explicit mental imagery, then the cuing effect might diminish or disappear when the position of the target is uncertain. Alternatively, if the facilitatory effect of auditory cues does not depend on overt imagery, then, (assuming mental imagery is position-specific), varying the stimulus position should not diminish the cuing effect. In Experiments 3 and 4, we compared the effect of cues on simple detection in cases where the position of the target stimulus was certain to when the stimulus had some position uncertainty. To further assess contributions of mental imagery, we obtained from each participant a measure of subjective visual imagery.

**Figure 4:**
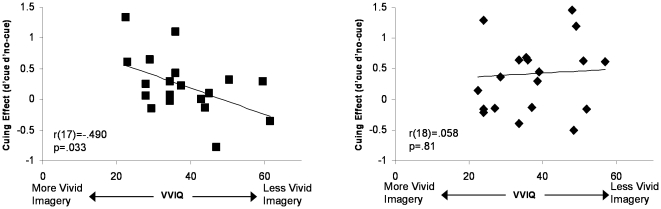
Results from Experiments 3 and 4. Left: Effects of auditory cues on the detection of cued visual objects versus objects cued with the uninformative word “ready” (Experiment 3). Right: Results from Experiment 4 in which the position of the to-be-detected stimuli was made unpredictable through random jitter. Bars indicate ±1 SE of the difference between the means.

The results of Experiments 3 and 4 mirrored those of Experiments 1 and 2. Detection performance on the cued trials was greater than performance on the non-cued trials (Table 1; Figure 4). As in Experiments 1–2, the sensitivity advantage arose from greater hit rates: in Experiment 3 auditory cues increased hit rates from .56 to .66, *t*(19) = 2.73, *p* = .013. An even more reliable cuing effect was obtained in Experiment 4. Cued trials produced significantly greater *d′* in both cases (Tables 3–4).

**Figure 5:**
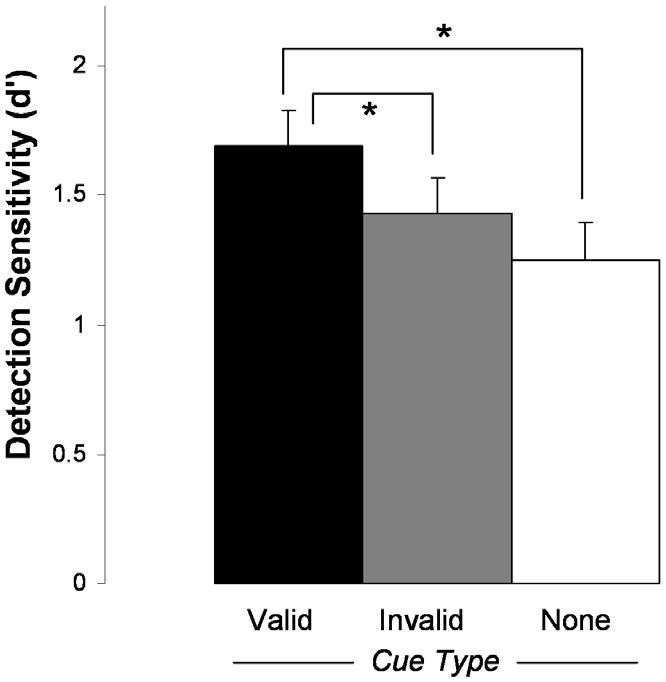
The magnitude of the cuing effect as a function of individuals' subjective rating of vividness of visual imagery. The relationship observed in Experiment 3 (left) disappears when the to-be-detected stimulus is presented with some spatial uncertainty, as in Experiment 4 (right).

The average imagery score was 38.01 (*SD* = 11.1). This score did not vary between Experiments 3 and 4, *t*<1, and did not correlate significantly with hit rates, false alarms, or *d′* on either cued or non-cued trials for either experiment (all *p*s>.3). However, in Experiment 3, with the position of the stimulus fixed at the center, imagery scores were significantly correlated with the size of the cuing effect (*d′*_cued-trials_–*d′*_uncued-trials_) (Figure 5-left). Individuals who scored as having the most vivid imagery (lowest VVI scores) were also the individuals who benefited most from hearing auditory labels, *r*(18) = −.490, *p* = .033 (VVQ data from one subject were missing due to experimenter error). As in Experiment 1, cuing also facilitated RTs, by 54 ms in Experiment 3, *t*(19) = 3.00, *p* = .007, and marginally in Experiment 4: 32 ms, *t*(19) = 1.79, *p* = .09.

Varying the position of the target (Experiment 4) did not reduce the facilitatory effect of auditory cues on object detection, but eliminated the correlation between imagery and the cuing effect: *r*(19) = .058 (Figure 5-right). Thus, a manipulation known to reduce the efficacy of mental imagery appeared to do so, as evident by the disappearance of an effect of individual differences in imagery on the magnitude of the cuing effect, but did not reduce the overall magnitude of the present cuing effect (Figure 4, right; Table 1).

### Experiment 5

This final experiment addresses a potential concern that the failure to find a benefit of visual cues in Experiment 1 arises from a difference in the time-course of visual and auditory cues. For example, it is possible that visual cues also facilitate simple detection, but their effect is no longer measurable 750 ms after the offset of the cue (the delay used in all the present studies). Experiment 5 tested this possibility by shortening the cue-to-target delay from 750 ms to 200 ms.

gA repeated-measures ANOVA revealed that performance was not affected by cuing, *F*<1. There was a marginal cuing×target-presence interaction, *F*(1,15) = 2.45, p = .14. Subsequent analyses showed that visual cues nonreliably increased hit-rates (Table 5), *t*(15) = 1.20, *p* = .25, but also (nonreliably) increased false-alarm rates, *t*(15) = 1.57, *p* = .14. There was no reliable difference in detection sensitivity (*d′*), *t*<1. There were also no effects of cuing on RTs, *F*<1. A cross-experiment comparison of the auditory cuing effect of Experiment 1 to the cuing effect in the present experiment found a significant difference between the two, *t*(34) = 2.04, *p*<.05, showing that auditory cues in Experiment 1 facilitated simple visual detection significantly more than visual cues in the present study. There was no reliable difference between overall performance in the present experiment and the visual condition of Experiment 1.

These results show that even when the delay between the cue and target is substantially reduced (from 750 ms to 200 ms), valid visual cues do not facilitate performance in a simple visual detection task.

### General Discussion

Being verbally informed of the identity of the target stimulus enhanced detection sensitivity of the named item. The possibility of a non-specific facilitatory effect of auditory stimulation was ruled out by Experiments 2 and 3. Experiment 2 contrasted valid and invalid cues: valid cues facilitated performance while invalid cues did not. Interestingly, the size of the cuing effect correlated with reports of vividness of mental imagery (Experiment 3): more vivid imagers showed the greatest auditory cuing benefits. When the position of target was jittered—a manipulation designed to make an explicit mental imagery strategy ineffective—individual measures of mental imagery no longer correlated with the cuing effect, which itself remained unchanged (Experiment 4). A further question concerns the specificity of the cuing effect. The results of Experiment 4 indicate that hearing a verbal cue enhances detection of the named object even if its exact position is unknown, suggesting that the effect induced by the auditory labels has a degree of position invariance. The present studies do not address a related question: what range of visual forms does hearing a label help detect, e.g., does hearing “emm” enhance detection of both uppercase and lowercase Ms?

Interestingly, although auditory verbal cues increased detection sensitivity, *visual* cues did not. This finding makes some sense when one considers that linguistic cues involve a non-overlapping format of sensory information that is globally statistically independent of the visual format of information in the detection task itself. By contrast, visual cues involve the same format of information as the detection task, and therefore do not provide converging sensory evidence from independent sources when the to-be-detected stimulus is presented. Experiment 5 showed that the failure to find improved detection following a visual cue was not due to an excessively long delay between the cue and the target (though it remains possible that visual cues would be effective in a presentation schedule not tested in the present work).

The auditory cues in the present studies were cuing orthographic forms (i.e., shapes). The present results of cuing effects on perceptual sensitivity thus contradict claims that perceptual sensitivity can be improved for spatial locations, but not for non-spatial features [14] (In contrast to Theeuwes and Van der Burg's task which involved searching through an array of multiple objects [14], in our task participants did not need to identify or categorize, but merely detect the presence of a single object).

It is possible that the failure to find effects of non-spatial cues on perceptual sensitivity is due to an exclusive focus on visual cues, which are, in fact, ineffective in improving visual sensitivity for non-spatial features. A finding that non-visual cues increase *d′* in a simple detection task is compatible with one of two broad conclusions: a) visual detection processes in visual cortex are influenced by auditory linguistic signals, or b) the process of detecting visual signals includes non-visual areas of cortex which are richly influenced by auditory linguistic signals. Either conclusion requires rejecting the assumption that “simple” visual tasks such as object detection depend only on the visual characteristics of a stimulus [i.e., that early vision is cognitively impenetrable, 36]. The present findings appear to conform to Pylyshyn's [36] requirements for evidence of cognitive penetrability of early vision because information from outside the visual system (the linguistic label) is affecting visual sensitity.

We conclude based on the present findings that auditory verbal cues actually alter perceptual processing of the named objects rather than alter a higher level decision process. Support for this conclusion comes from two sources: First, we observed changes in perceptual sensitivity (*d′*) but not in criterion. Second, contrary to a decision-level account, although visual cues and verbal cues both delivered the same letter-identity information, only the verbal cues enhanced detection.

The observed findings may be thought of as a type of priming, albeit in a different sense from the way priming is usually discussed. Priming as classically defined involves the spreading of activation among semantic and conceptual representations and does not necessarily entail an account in which a linguistically-primed object representation influences the operation of putatively lower-level processes involved in the visual detection of that same object. The present findings are thus incompatible with strictly bottom-up models of priming. Several contemporary theories of repetition priming, however, do rely on feedback (e.g., modulation of posterior cortical regions by anterior regions) [37]. Such feedback is necessary to explain why the onset of many repetition priming effects in more posterior regions (e.g., ventral cortex) is observed only after frontal activity [38]. The present findings are consistent with models of priming that incorporate top-down feedback and the framework of vision as prediction e.g., [39].

Another key differences between the present results and those typically obtained in the priming literature is the short-lived timecourse of the cue-induced enhancement we observe. Perceptual priming is typically long-lasting [40]: priming a stimulus can facilitate its *identification* for weeks. In contrast, cuing a stimulus with its auditory label facilitated its *simple detection* only for the duration of the trial. Although the present studies were not designed to measure the timecourse of the cuing effect, we can infer that enhanced target detection due to the prime did not last for much longer than a single trial, otherwise performance on the intermixed cuing and non-cuing trials would converge.

Another difference between the present phenomenon and that of perceptual priming is that perceptual priming is highly sensitive to such physical manipulations as changes in typography between the prime and test stimuli [41], [42]. In the present studies, the cue and the to-be-detected stimulus were presented in different modalities—a manipulation arguably much more significant than a change of font. When the cue and the to-be-detected-stimulus were presented in the same modality (visual condition of Exp. 1 and Exp 5), the cue did not affect detection performance—an finding not predicted by a bottom-up perceptual priming account.

Related to the present findings are findings showing an effect of visual input, namely lip movements, on speech perception and spoken word recognition, e.g., [43], [44]. For example when a spoken word stimulus is immersed in enough noise that correct identification is near-threshold, the influence of a second modality (visual input of lips moving) has its maximal influence on accuracy [45]. Moreover, neuroimaging work has shown that viewing lip movements influences the pattern of activity in auditory cortex [46].

One way to understand our results is by conceiving of verbal labels as providing modulatory feedback to the visual system (The Label Feedback Hypothesis) [8], [47]. Attention (one form of top-down control) has been shown to affect response properties of neurons in the very first visual area receiving top-down projections—the lateral geniculate nucleus [48]—and there is a large literature on effects of context, task-demands, and expectations on neural responses in primary visual cortex, see [49] for review. The present results offer evidence that verbal labels, by virtue of their pre-existing association with visual stimuli, modulate visual processing by providing a “head-start” to the visual system, facilitating the processing of stimuli associated with the label. This type of continuous interaction between top-down and bottom-up processes is consistent with a number of theoretical frameworks [50]–[52].

In summary, the present findings indicate that a linguistic cue in the form of a letter name makes an otherwise invisible letter visible. In contrast, a visual preview of the target stimulus does not lead to a detection enhancement, indicating that verbal cues are especially effective in enhancing visual detection. These studies inform our understanding of how language—a uniquely human trait—interacts with a visual system that we largely share with other primates. Currently ongoing experiments indicate that similar results can be obtained for pictures of everyday objects and animals: hearing common nouns can facilitate the detection of pictures from the named category [53].

Many unanswered questions remain: First, does the cuing effect generalize to more complex objects? Because the cuing effect was observed in a design that intermixed cued and uncued trials, the cue-induced facilitation must be transient component, but its duration and temporal profile are at present unknown. Second, how general are the present findings of a cross-modality advantage for visual detection? Future work will need to explore whether the cross-modality advantage is present in the reverse direction: is detection of an *auditory* target improved more by a visual cue than a corresponding auditory cue? Based on the present results, the answer is unclear, however, ongoing studies, Lupyan and Thompson-Schill [54] suggest that the format of the cue, in addition to its modality, is important: verbal auditory cues (e.g., “cow”) facilitated visual identification and discrimination more than nonverbal auditory cues (e.g., the sound of a cow mooing”). Finally, future research will need to investigate the process by which learning to associate new labels with new stimuli enhances detection of these stimuli. Such work may inform our understanding of how, and to what degree, learning different languages can induce differences in perceptual processing [54]–[56].

## Materials and Methods

### Participants

A total of 80 Cornell University undergraduates and 16 University of Pennsylvania undergraduates, ages 18–22, volunteered in five Experiments in exchange for course credit: 40 in Experiment 1, 10 in Experiment 2, 20 each in Experiments 3–4, and 16 in Experiment 5. All were naïve to the hypothesis and none participated in more than one study. *Ethics statement*. The studies were conducted in strict compliance with the IRBs of Cornell University and University of Pennsylvania. The IRBs of both universities approved the described studies. Written consent was obtained for each participant.

### Materials

The stimuli were uppercase English letters, rendered using the Arial font and subtended approximately 2.2° (Vertical)×1.8° (Horizontal) visual-angle. Letters were chosen as stimuli because of the strong pre-existing associations between their visual forms and their names. The letters used in the main part of the experiment were: B,E,F,H,M,O,R,U,V,Y. The visual cues were identical to the stimuli to-be-detected. The auditory cues were pre-recorded letter names, obtained from an online repository: http://community.voxeo.com/library/audio/prompts/alphabet/index.jsp. The letter names, as recorded, were approximately 650 ms in duration.

### General Procedure

The participants' task was to detect uppercase letters, and respond *present* if they saw an object, and *absent* if they thought only the mask was present (Figure 1). On exactly half of the trials, a cue preceded the detection task allowing us to study the effect of the cue on detection performance. The *auditory* and *visual* conditions differed only in what happened during this cuing part of the trial. In the *visual* condition, a letter cue was presented on half of the trials alerting the participants to the identity of the to-be-detected stimulus. On the remaining trials, the fixation cross was replaced by a gray square for a duration identical to the cue duration (650 ms). The auditory condition was identical except the cue was auditory, consisting of the letter name of the to-be detected letter (e.g., “emm” for M). Participants were told that the cue would predict the identity of the to-be-detected letter, but not its presence (cf. Experiment 2 in which the cue did *not* predict the identity of the letter). During the presentation of the auditory cue, the fixation cross was replaced by a gray square for 650 ms. The display then reverted back to the fixation cross for 750 ms after which the detection part of the trial began. On exactly half of the trials a faint uppercase letter was flashed for 53 ms and then masked by randomly oriented line segments. On the remaining half of the trials, no letter was present during this interval. The mask for each trial was selected randomly from 100 random masks generated for each participant. This ensured that participants could not anticipate the perceptual details of the mask.

To observe the effect of the cue on object detection, the task had to be difficult enough to avoid ceiling-level performance. Pilot work revealed that participants were able to detect white-on-black letters even when they were presented for one screen refresh (13.3 ms). We thus adjusted the contrast of the letter stimuli for each participant by using an informal staircasing procedure during which the contrast of the to-be-detected stimulus was lowered following a correct response and increased following an incorrect response (the two directions were interleaved). The contrast step-size decreased every 20 trials.

Each experimental session began with the staircasing procedure starting with plainly visible letters, and lasting 75 trials. The first 15 trials were considered practice and used accuracy feedback—a buzz sounded after incorrect responses. There were no cues used during staircasing and all 26 letters were used as stimuli. The procedure was designed to produce hit rates of approximately 55%.

The main part of the experiment consisted of 5 blocks of 40 trials (stimulus-present vs. stimulus-absent × cue vs. no cue × stimulus identity). Trial order was random with the target present on exactly half of the trials. On exactly half of the target-present trials, the target was preceded by a cue. Participants gave 2-alternative target present/absent responses using a gamepad controller. Responses were counted as hits if a ‘present’ response followed a presented letter stimulus, and as ‘false alarms’ if it followed an absent stimulus. Hand-to-response mapping was counterbalanced between participants.

### Experiments 3–4

These experiments was identical to the auditory-cue condition of Experiment 1 except no-cue trials now included the uninformative auditory cue “ready” which equated general auditory arousal across trial types. In Experiment 4, the to-be-detected stimulus was displayed with some spatial uncertainty—its position was randomly jittered by 0.5°–1.5° horizontally and vertically (measured from fixation to the center of the letter). Following both experiments, participants completed a vividness of visual imagery (VVI) questionnaire [57] which contained 16 imagery questions to be completed once with eyes open, and once with eyes closed. The dependent measure was the average score of eyes-open and eyes-closed conditions, ranging from a minimum VVI score of 16 (all responses: “Perfectly clear and as vivid as normal vision”) to a maximum of 80 (all responses: “No image at all, you only ‘know’ that you are thinking of the object”).

### Experiment 5

The procedure was identical to the visual-cue condition of Experiment 1 except the 750 ms delay between the end of the cuing period and the onset of the to-be-detected stimulus was reduced to 200 ms. Reducing the delay further risked that participants would confuse the cue itself for the target stimulus.
