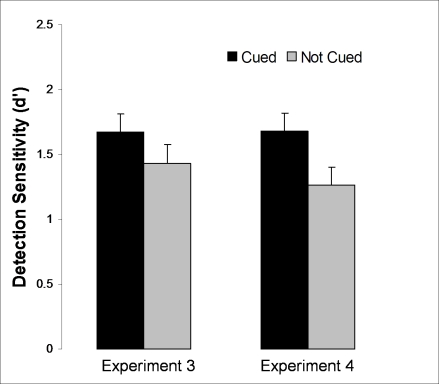# Correction: Making the Invisible Visible: Verbal but Not Visual Cues Enhance Visual Detection

**DOI:** 10.1371/annotation/9b8741e2-0f5f-49f9-9eaa-1b0cb9b8d25f

**Published:** 2010-08-25

**Authors:** Gary Lupyan, Michael J. Spivey

Figure 4 was published with an error in the labels under the bars. The labels should read Experiment 3 / Experiment 4 instead of Experiment 2 / Experiment 3. Please see the corrected figure 4 here: 

**Figure:**