# Correction: Making the Invisible Visible: Verbal but Not Visual Cues Enhance Visual Detection

**DOI:** 10.1371/annotation/0eabb45a-f9da-4c9d-8555-0efee6e777f8

**Published:** 2010-08-25

**Authors:** Gary Lupyan, Michael J. Spivey

The images for figures 3, 4, and 5 were incorrectly switched. The image that appears as figure 3 should be figure 4, the image that appears as figure 4 should be figure 5, and the image that appears as figure 5 should be figure 3. 

